# Decreased mitochondrial D-loop region methylation mediates an increase in mitochondrial DNA copy number in CADASIL

**DOI:** 10.1186/s13148-021-01225-z

**Published:** 2022-01-04

**Authors:** Jiewen Zhang, Junkui Shang, Fengyu Wang, Xuejing Huo, Ruihua Sun, Zhixia Ren, Wan Wang, Miaomiao Yang, Gai Li, Dandan Gao, Ruijie Liu, Pingping Bai, Shuyi Wang, Yanliang Wang, Xi Yan

**Affiliations:** 1 Department of Neurology, Henan Provincial People’s Hospital, Zhengzhou University People’s Hospital, Henan University People’s Hospital, Zhengzhou, 450003 Henan China; 2 Department of Health Management, Henan Key Laboratory of Chronic Disease Management, Henan Provincial People’s Hospital, Zhengzhou University People’s Hospital, Henan University People’s Hospital, Zhengzhou, 450003 Henan China; 3 Department of General Practice, Henan Provincial People’s Hospital, Zhengzhou University People’s Hospital, Henan University People’s Hospital, Zhengzhou, 450003 Henan China; 4 Department of Nephrology, Henan Provincial Key Laboratory of Kidney Disease and Immunology, Henan Provincial People’s Hospital, Zhengzhou University People’s Hospital, Henan University People’s Hospital, Zhengzhou, 450003 Henan China

**Keywords:** CADASIL, Mitochondrial D-loop region, Mitochondrial DNA copy number, Methylation

## Abstract

**Background:**

Cerebral autosomal dominant arteriopathy with subcortical infarcts and leukoencephalopathy (CADASIL) is a typical neurodegenerative disease associated with mitochondrial dysfunction. Methylation of the D-loop region and mitochondrial DNA copy number (mtDNAcn) play a critical role in the maintenance of mitochondrial function. However, the association between D-loop region methylation, mtDNAcn and CADASIL remains unclear.

**Methods:**

Overall, 162 individuals were recruited, including 66 CADASIL patients and 96 age- and sex-matched controls. After extracting genomic DNA from the peripheral white blood cells, levels of D-loop methylation and mtDNAcn were assessed using MethylTarget sequencing and real-time PCR, respectively.

**Results:**

We observed increased mtDNAcn and decreased D-loop methylation levels in CADASIL patients compared to the control group, regardless of gender stratification. Besides, we found a negative correlation between D-loop methylation levels and mtDNAcn. Mediation effect analysis shows that the proportion of the association between mtDNAcn and CADASIL that is mediated by D-loop methylation is 11.6% (95% CI 5.6, 22.6). After gender stratification, the proportions of such associations that are mediated by D-loop methylation in males and females were 7.2% (95% CI 2.4, 19.8) and 22.0% (95% CI 7.4, 50.1), respectively.

**Conclusion:**

Decreased methylation of the D-loop region mediates increased mtDNAcn in CADASIL, which may be caused by a compensatory mechanism of mitochondrial dysfunction in patients with CADASIL.

**Supplementary Information:**

The online version contains supplementary material available at 10.1186/s13148-021-01225-z.

## Introduction

Cerebral autosomal dominant arteriopathy with subcortical infarcts and leukoencephalopathy (CADASIL), the most common hereditary cerebral small vessel disease, is caused by mutations in the *NOTCH3* gene. CADASIL is a typical neurodegenerative disease that is characterized by a progressive loss of vascular smooth muscle cells (VSMCs) [1]. Mitochondria is an important organelle that controls cellular energy metabolism of cells [2]. Mitochondrial dysfunction is the precursor of pathological changes in the majority of neurodegenerative disease [3, 4]. Several studies have suggested a close association between mitochondrial dysfunction and VSMCs loss in CADASIL [5–7]. In fact, one study demonstrated that the number of mitochondria in VSMCs among patients with CADASIL was increased, and most of them were abnormal [8]. In addition, measurement of mitochondrial membrane potential suggested that the percentage of fully functional mitochondria in VSMCs of patients with CADASIL was lower than that in the controls. These findings indicate that the alteration of mitochondrial function in VSMCs among patients with CADASIL may have an effect on the pathologically important cellular functions [8]. However, there is little epidemiological evidence that connects mitochondria with CADASIL. Therefore, exploring the epidemiological association between mitochondria and CADASIL is necessary to further understand the occurrence and development of CADASIL.

The maintenance of mitochondrial function is largely dependent on the normal expression of mitochondrial proteins, which are encoded by mitochondrial DNA (mtDNA) [9]. Due to a lack of histone protection, mtDNA is more susceptible to the genetic and environmental factors than nuclear DNA [10]. In addition, due to a lack of ability to repair itself, mtDNA is usually degraded after being damaged, and its function is usually compensated by newly synthesized mtDNA, which may cause an abnormal reduction or increase of mtDNA copy number (mtDNAcn). Therefore, mtDNAcn is generally considered to be an important biomarker of mitochondrial function. It is also the most commonly used substitute for mitochondrial function in population studies [11]. The study of changes in mtDNAcn, which is often used to evaluate the condition of patients from the perspective of mitochondrial function, has important clinical significance [12].

It is well known that mtDNA contains a noncoding region, known as the D-loop region, which regulates the transcription and replication of mtDNA [13]. Recent evidence suggests that epigenetic modification of the mitochondrial genome can contribute to neurodegeneration [14]. Demethylation of the D-loop region and elevated mtDNAcn have been observed in both sporadic amyotrophic lateral sclerosis (ALS) patients and carriers of ALS-linked *SOD1* mutations, which suggests that D-loop methylation may negatively regulate mtDNAcn [15, 16]. Notably, the abnormal level of D-loop methylation is often observed among other degenerative diseases. Specifically, D-loop methylation levels become dysregulated in animal models of Alzheimer's disease (AD) [17, 18]. In peripheral blood DNA among patients with late-onset AD, there is a decreased methylation of the D-loop region [19]. Additionally, D-loop methylation levels in the substantia nigra were also significantly reduced in post-mortem patients with Parkinson's disease (PD), compared to healthy controls [17]. Considering that CADASIL is also a typical neurodegenerative disease, we speculate that the level of D-loop methylation among patients with CADASIL may change. However, it is still lack of necessary evidence and therefore urgently needs to be further studied.

Given this, we speculate that there may be a complex association between mtDNAcn, D-loop methylation and CADASIL. Herein, we conducted a case–control study, quantified DNA methylation levels of the D-loop region and analyzed mtDNAcn in the peripheral blood leukocytes, in order to confirm this hypothesis and provide epidemiological evidence of the relationship between mitochondrial function and CADASIL.

## Materials and methods

### Study population

We recruited 162 participants for this study, including 66 patients with CADASIL and 96 participants without neurological symptoms and signs. CADASIL patients were screened with *NOTCH3* mutations among patients with clinical and neuroimaging evidences suggestive of cerebral small vessel disease. All included patients with CADASIL met established diagnostic criteria [20, 21] and were diagnosed by neurologists at the Henan Provincial People’s Hospital. Patients were excluded if they had incomplete clinical information or white matter lesions that were caused by other clear causes, including poisoning, metabolism disorder, demyelination, infection and immune dysfunction. In addition, among the 96 recruited controls, age and gender were matched to patients, but not relatives of the CADASIL patients recruited in this study. Prior to being included in the study, all participants were well informed about the study purpose and procedures and provided written informed consent. All research procedures were granted approval by the Ethics Committee of Henan Provincial People’s Hospital.

### Collection of general data and biological samples

Trained investigators conducted face-to-face interviews with participants in order to collect general data, including age, gender, history of hypertension, diabetes mellitus and dyslipidemia. In addition, fasting peripheral blood samples of each participant were collected by nurses at the Henan Provincial People’s Hospital. The blood was then centrifuged (1500× *g* for 15 min) immediately in order to separate the plasma and white blood cells. White blood cells were stored at − 80 °C until further analysis.

### Detection of mtDNAcn

Whole blood genomic DNA extraction kit (GK1042, Generay Biotech, China) was utilized to extract genomic DNA among white blood cells. Next, a spectrophotometer (NanoDrop 1000, Thermo, Waltham, MA, USA) was utilized to quantify the concentrations of the extracted DNA samples.

The circulating mtDNA content in the peripheral blood was determined as the ratio of two mitochondrial regions to two single-copy nuclear control genes by real-time polymerase chain reaction (PCR) analysis (ABI 7900, Applied Biosystems, USA). In brief, mitochondrial-encoded NADH dehydrogenase 1 (MTND1) and mitochondrial-encoded NADH dehydrogenase 5 (MTND5) were chosen as mitochondrial genes. *SLCO2B1* and *SERPINA* were selected as single-copy nuclear genes. The real-time PCR conditions were as follows: 1 min at 95 °C, followed by 40 cycles of 95 °C for 10 s and 60 °C 31 s. The absence of primer dimers and amplification specificity were validated by melting curve analysis (1 min at 95 °C, 30 s at 60 °C, 30 s at 95 °C) at the end of each run. The detailed PCR are shown in Additional file 1: Table S1. The cycle threshold (Ct) value is defined as the number of cycles at which the fluorescence of the amplified product of the corresponding gene or DNA fragment is greater than the fixed threshold of blank control. In this study, we used the system default value as the threshold and the final Ct values are calculated as the average of triplicates. Relative quantification of the copy number of mtDNA using nuclear DNA as a reference was determined using the following formula: [2^(Ct_SLCO2B1_ – Ct_MTND1_) + 2^(Ct_SERPINA_ – Ct_MTND5_)]/2.

### D-loop methylation analysis

The methylation levels of the D-loop region were determined by MethylTarget sequencing. In brief, DNA samples were treated with sodium bisulfite through the use of EZ DNA Methylation-Gold™ Kit (Zymo Research, CA, USA), according to manufacturer's instructions. Subsequently, in order to completely identify the methylation levels of all CpG sites that are located in the D-loop region, we divided the D-loop into two fragments and performed net-PCR with optimized primers (Forward primer 1: TAT TAT AGT GAG AAT TTT ATG ATG GAT TATGT, Reverse primer 1: CCC TAA TTC CCC CCA TCCT; Forward primer 2: GGG TAT GAG TTA GTA GTT TTT GTG AGTTT, Reverse primer 2: CCA CAT TAA CAA CAT AAA ACC CTCAT), in order to amplify the targeted DNA sequence. Then, the targeted DNA fragments were sequenced using the Illumina Hiseq 2000 platform, according to the manufacturer's protocols.

### Statistical analyses

Two independent samples *t* test and Chi-square test were used to analyze the difference in the characteristics between the case and control group. Besides, after treating the median of each tertile of D-loop methylation levels as a continuous variable, we performed a linear trend across increasing tertiles of D-loop methylation levels and mtDNAcn. After determining the linear association, multiple linear regression models were performed to evaluate the association between CADASIL, D-loop methylation levels and mtDNAcn. In addition, a mediation effect analysis of the statistical analysis system (SAS) macros described by Lin et al. [22] was utilized to evaluate the association among CADASIL, D-loop methylation levels and mtDNAcn. The mediation effect analysis method, which was developed by the Harvard T. H. CHAN School of Public Health, can be found at the website of http://www.hsph.harvard.edu/donna-spiegelman/software/mediate/ [22].

The SAS statistical software package (version 9.4, SAS Institute Inc., Cary, NC, USA) was utilized for data analyses. The *P* values were bilateral, and the significance level was 0.05.

## Results

### Characteristics of the study population

The characteristics of all participants, which included 66 patients with CADASIL and 96 controls, are summarized in Table 1. There was no significant difference in the average age between the case group (55.68 years) and control group (55.50 years) (*P* = 0.917). Furthermore, we did not find any significant difference in the ratio of males (*P* = 0.834), hypertension (*P* = 0.529), diabetes mellitus (*P* = 0.216) and dyslipidemia (*P* = 0.118) between the case group and the control group.

**Table 1:** Characteristics of the study population

Characteristic	CADASIL	Controls	*t*/*χ*^2^	*P*
*n*	66	96		
Age	55.68 ± 11.06	55.50 ± 10.79	0.104	0.917
Gender			0.044	0.834
Male	43 (65.15%)	61(63.54%)		
Female	23 (34.85%)	35 (36.46%)		
Hypertension			0.397	0.529
Yes	28 (42.42%)	36 (37.50%)		
No	38 (57.58%)	60 (62.50%)		
Diabetes mellitus			1.552	0.216
Yes	13 (19.70%)	12 (12.50%)		
No	53 (80.30%)	84 (87.50%)		
Dyslipidemia			2.449	0.118
Yes	7 (10.61%)	19 (19.79%)		
No	59 (89.39%)	77(80.21%)		

### mtDNAcn differences between CADASIL group and control group

**Fig. 1:**
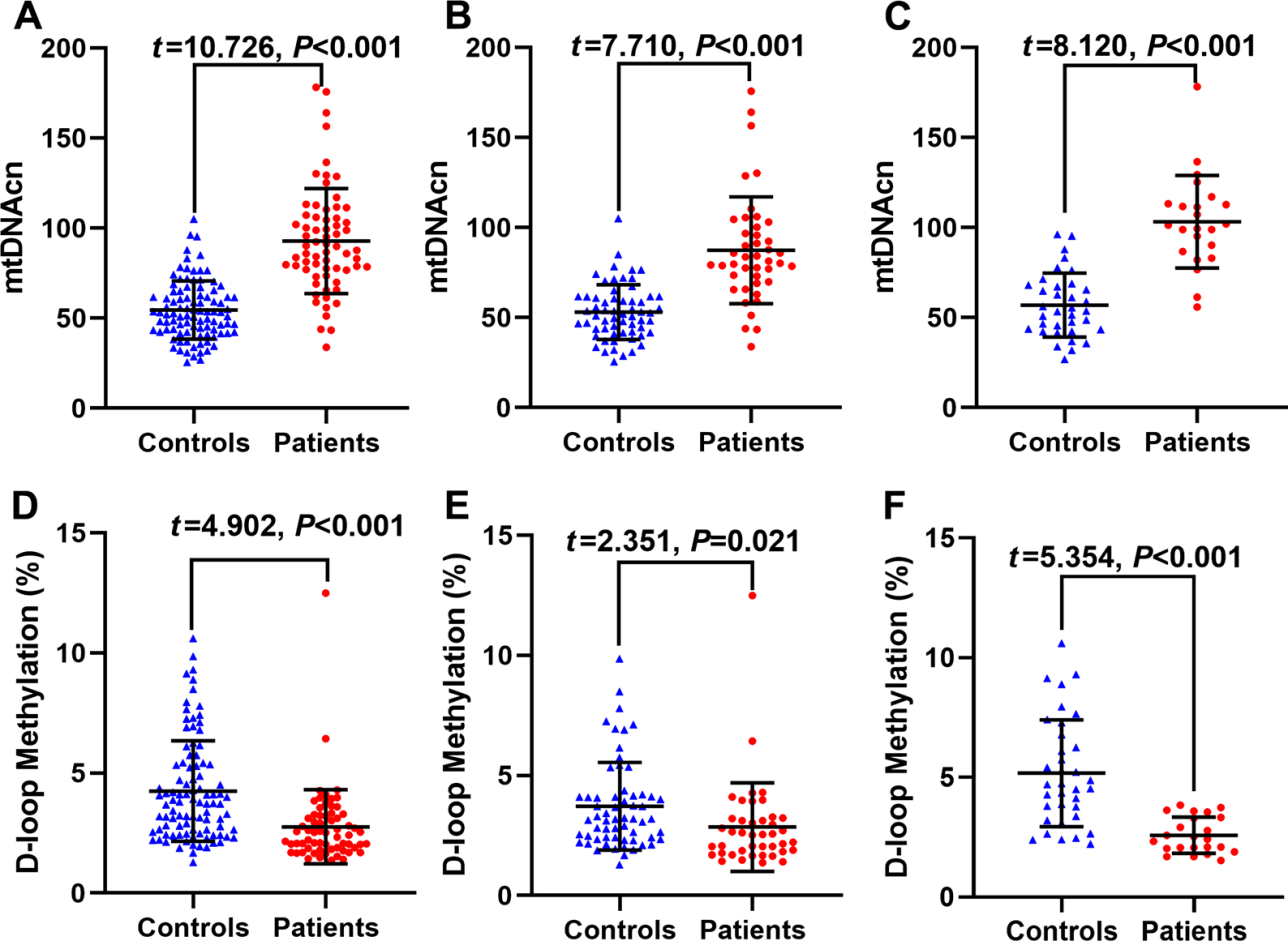
The differences of mtDNAcn and D-loop methylation levels between the CADASIL group and the control group. **A** In the total population, the mtDNAcn of the case group (92.94 ± 29.19) is higher than that of the control group (54.53 ± 16.18) (*P* < 0.001). **B** In the male population, mtDNAcn of the case group (87.42 ± 29.71) is higher than the control group (53.12 ± 15.19) (*P* < 0.001). **C** In the female population, mtDNAcn of the case group (103.27 ± 25.73) is higher than the control group (56.99 ± 17.73) (*P* < 0.001). **D** In the total population, D-loop methylation of the case group (4.25% ± 2.10%) is higher than the control group (2.76% ± 1.55%) (*P* < 0.001). **E** In the male population, D-loop methylation of the case group (2.85% ± 1.84%) is lower than the control group (3.72% ± 1.83%) (*P* = 0.021). **F** In the female population, D-loop methylation is lower in the case group (2.59% ± 0.75%) than in the control group (5.18% ± 2.23%) (*P* < 0.001). All tests were performed using two independent sample *t* tests: long bar for mean and short bar for standard deviation

Two independent sample *t* tests show that the mtDNAcn of the case group (92.94 ± 29.19) is higher than that of the control group (54.53 ± 16.18) (*P* < 0.001) in the total population. Specifically, in the male population, mtDNAcn of the case group (87.42 ± 29.71) was higher than that of the control group (53.12 ± 15.19) (*P* < 0.001). In the female population, mtDNAcn of the case group (103.27 ± 25.73) was also higher than the control group (56.99 ± 17.73) (*P* < 0.001) (Fig. 1).

Multiple linear regression analysis shows that, with the occurrence of CADASIL, mtDNAcn increased by 38.53 units (95% CI 31.67–45.38, *P* < 0.001). In particular, after adjusting for age, hypertension, diabetes mellitus and dyslipidemia, the mtDNAcn in male and female population with CADASIL was increased by 34.41 (95% CI 25.80–43.02, *P* < 0.001) and 48.47 units (95% CI 36.65–60.29, *P* < 0.001), respectively (Table 2).

**Table 2:** The differences of mtDNAcn between CADASIL group and control group

	mtDNAcn			
	Crude *β *(95% CI)	*P*	Adjusted *β* (95% CI)	*P*^a^
Total	38.41 (31.43,45.38)	< 0.001	38.53 (31.67,45.38)	< 0.001
Male	34.30 (25.66,42.93)	< 0.001	34.41 (25.80,43.02)	< 0.001
Female	46.28 (35.30,57.25)	< 0.001	48.47 (36.65,60.29)	< 0.001

^a^Adjust for gender, age, hypertension, diabetes mellitus, dyslipidemia

Sensitivity analysis indicated that the status of CADASIL still affects mtDNAcn with *β* values ranging from − 1.49 to − 1.45, after adjusting for different confounders in the total population (all *P* < 0.001). After stratification by gender, we observed that the status of CADASIL can affect mtDNAcn after adjusting for different confounders, with *β* values ranging from − 0.90 to − 0.85 in males (all *P* < 0.05) and ranging from − 2.86 to − 2.70 in females (all *P* < 0.001) (Additional file 1: Table S2).

### Differences in D-loop region methylation levels between the CADASIL group and control group

Two independent sample *t* tests show that, in the total population, D-loop methylation of the case group (4.25% ± 2.10%) is significantly higher than that of the control group (2.76% ± 1.55%) (*P* < 0.001). In the male population, D-loop methylation of the case group (2.85% ± 1.84%) is significantly lower than that of the control group (3.72% ± 1.83%) (*P* = 0.021). Similarly, in the female population, D-loop methylation is significantly lower in the case group (2.59% ± 0.75%) compared to the control group (5.18% ± 2.23%) (*P* < 0.001) (Fig. 1).

Multiple linear regression analysis showed that, after adjusting for potential confounders, methylation levels in the D-loop region were found to decrease by 1.46% (95% CI 2.04–0.87, *P* < 0.001) in the total population with the occurrence of CADASIL. The methylation levels of the D-loop region in the male and female population were decreased by 0.88% (95% CI 1.60–0.16, *P* = 0.017) and 2.86% (95% CI 3.88–1.84, *P* < 0.001), respectively, with the occurrence of CADASIL (Table 3).

**Table 3:** The differences of D-loop region methylation levels between CADASIL group and control group

	D-loop region methylation			
	Crude *β *(95% CI)	*P*	Adjusted *β* (95% CI)	*P*^a^
Total	− 1.49 (− 2.08,− 0.90)	< 0.001	− 1.46 (− 2.04,− 0.87)	< 0.001
Male	− 0.86 ( − 1.58,− 0.15)	0.018	− 0.88 (− 1.60,− 0.16)	0.017
Female	− 2.59 ( − 3.52,− 1.66)	< 0.001	− 2.86 (− 3.88,− 1.84)	< 0.001

^a^Adjust for gender, age, hypertension, diabetes mellitus, dyslipidemia

Sensitivity analysis demonstrated that the status of CADASIL still affects D-loop methylation levels, with *β*s ranging from 38.44 to 38.63, after adjusting for different confounders in the total population (all *P* < 0.001). After stratification by gender, we observed that the status of CADASIL has an effect on the D-loop methylation levels after adjusting for different confounders, with *β*s ranging from 33.92 to 34.41 in males (all *P* < 0.05) and from 46.44 to 48.83 in females (all *P* < 0.001) (Additional file 1: Table S3).

### Associations between mtDNAcn and D-loop methylation

**Fig. 2:**
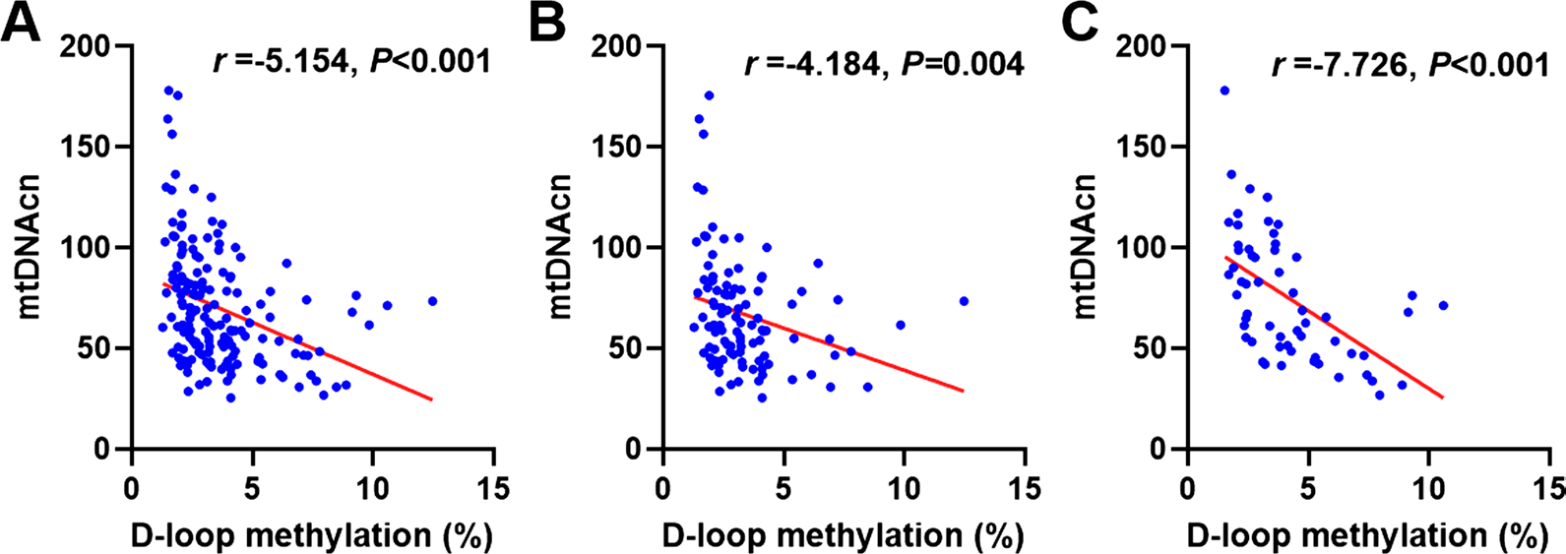
Associations between mtDNAcn and D-loop methylation levels. **A** In the total population, mtDNAcn is negatively correlated with D-loop methylation (*r* =  − 5.154, *P* < 0.001). **B** In the male population, mtDNAcn is negatively correlated with D-loop methylation (*r* =  − 4.184, *P* = 0.004). **C** In the female population, mtDNAcn is negatively correlated with D-loop methylation (*r* =  − 7.726, *P* < 0.001). All tests were performed by Pearson correlation analysis

Pearson correlation analysis indicates that the mtDNAcn is negatively correlated with methylation levels in the D-loop region, regardless of gender stratification (Fig. 2). Trend tests indicated that there is a linear association between methylation of D-loop region and mtDNAcn. Specifically, mtDNAcn increased by 5.15 units for each 1% decrease in the D-loop region methylation levels in the total population (*P* < 0.001). After adjusting for confounders, mtDNAcn increased by 5.74 units per 1% increase in methylation levels in the D-loop region (*P* < 0.001). After gender stratification, we observed that after adjusting for confounders, mtDNAcn increased by 4.32 units for every 1% decrease of methylation levels in the D-loop region in males (*P* = 0.019). In the female population, after adjusting for confounders, mtDNAcn increased by 7.65 units for every 1% decrease in methylation levels of the D-loop region (*P* < 0.001) (Table 4).

**Table 4:** Associations between mtDNAcn and D-loop methylation levels

D-loop methylation	mtDNAcn			
	Crude *β* (95% CI)	*P*	Adjusted *β *(95% CI)	*P*^a^
Total				
Tertile 1	Reference		Reference	
Tertile 2	− 12.23 (− 22.71,− 1.75)	0.022	− 14.39 (− 24.35,− 4.42)	0.005
Tertile 3	− 8.79 (− 11.99,− 5.59)	< 0.001	− 9.11(− 12.33,− 5.88)	< 0.001
* P* for Trend		< 0.001		< 0.001
Continuous	− 5.15 (− 7.24,− 3.07)	< 0.001	− 5.74 (− 7.80,− 3.69)	< 0.001
Male				
Tertile 1				
Tertile 2	− 15.86 (− 24.66,− 4.07)	< 0.001	− 16.61(− 25.28,− 7.95)	< 0.001
Tertile 3	− 10.01(− 15.62,− 4.41)	0.001	− 10.65 (− 16.10,− 5.19)	< 0.001
* P* for Trend		< 0.001		< 0.001
Continuous	− 4.18 (− 6.95,− 1.42)	0.003	− 4.32 (− 7.04,− 1.60)	0.019
Female				
Tertile 1				
Tertile 2	− 17.03 (− 29.46,− 4.60)	0.007	− 19.55 (− 32.96,− 6.14)	0.004
Tertile 3	− 9.99 (− 13.70,− 6.28)	< 0.001	− 10.44 (− 14.08,− 6.81)	< 0.001
* P* for Trend		< 0.001		< 0.001
Continuous	− 7.73 (− 10.77,− 4.68)	< 0.001	− 7.65 (− 10.56,− 4.74)	< 0.001

^a^Adjust for gender, age, hypertension, diabetes mellitus, dyslipidemia

Sensitivity analysis demonstrated that the methylation levels of D-loop still affect mtDNAcn, with *β*s ranging from − 5.77 to − 5.21, after adjusting for different confounders in the total population (all *P* < 0.001). After stratification by gender, we observed that the methylation levels of D-loop have an effect on the mtDNAcn after adjusting for different confounders, with *β*s ranging from − 4.32 to − 4.25 in males (all *P* < 0.05) and from − 7.74 to − 7.64 in females (all *P* < 0.001) (Additional file 1: Table S4).

### Mediation effect analysis of the estimated effect of D-loop methylation levels on regulating mtDNAcn changes in CADASIL

**Fig. 3:**
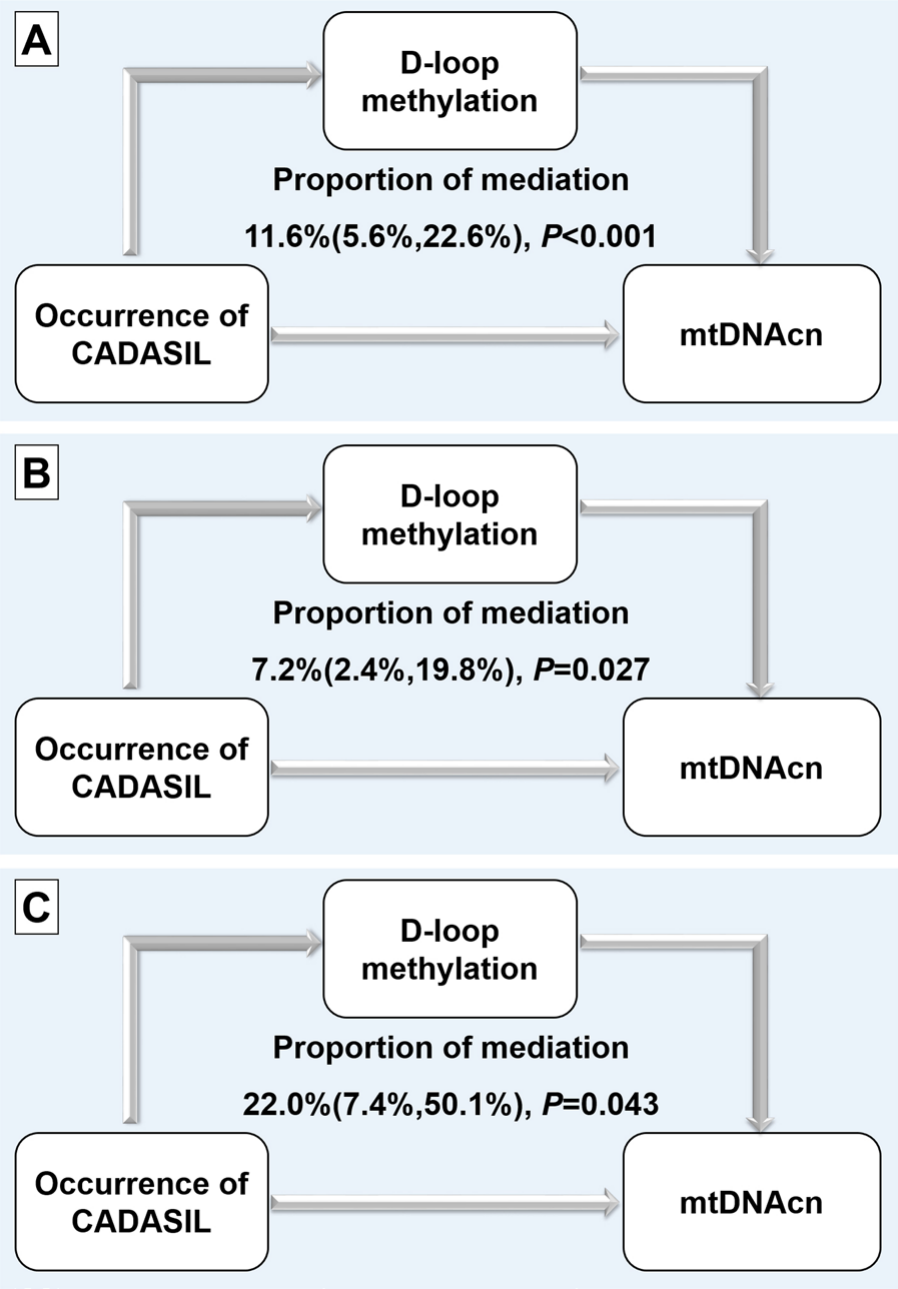
Mediation analysis for the role of D-loop methylation on mtDNAcn in CADASIL. Mediation analysis for the role of D-loop methylation changes in the associations between CADASIL and mtDNAcn in the total population (**A**), in males (**B**) and females (**C**) after adjusted for age, gender, hypertension, diabetes mellitus and dyslipidemia

The proportion of the associations between mtDNAcn and CADASIL mediated by D-loop methylation was 11.6% (95% CI 5.6, 22.6) (Fig. 3). After stratification of people according to gender, we observed that the alteration of D-loop methylation could still mediate the association between mtDNAcn and CADASIL. In addition, the proportions mediated by D-loop methylation in males and females were 7.2% (95% CI 2.4, 19.8) and 22.0% (95% CI 7.4, 50.1), respectively (Fig. 3).

## Discussion

In our study, we investigated mtDNAcn and methylation levels of the mitochondrial D-loop region in the peripheral blood of patients with CADASIL and controls. A significant increase in mtDNAcn and a decreased tendency in the D-loop region methylation levels were observed in patients with CADASIL, compared to controls. Furthermore, we observed a negative correlation between D-loop region methylation level and mtDNAcn. In addition, mediation effect analysis revealed that decreased mitochondrial D-loop region methylation level partly mediates the association between mtDNAcn and the occurrence of CADASIL.

Previous studies have discovered that the activity of mitochondrial respiratory complex I was significantly decreased in muscle biopsy of CADASIL patients [5]. They also determined that a lethal mutation of the *Notch* gene, *N55e*^*11*^, in Drosophila decreases activity of the mitochondrial respiratory complexes I and V, indicating that pathophysiology of CADASIL could include a defect in oxidative phosphorylation and mitochondrial respiratory chain activity responds to the Notch signaling pathway, directly or indirectly [5]. Additionally, muscle biopsies from CADASIL patients also indicated signs of mitochondrial accumulation and paracrystalline mitochondrial inclusions [6, 7]. Furthermore, an increased number of mitochondria in CADASIL VSMCs have been observed [8].

In the current study, we observed an increase in mtDNAcn in patients with CADASIL, suggesting that mitochondrial dysfunction likely exists in patients with CADASIL, which strengthens evidence provided by previous studies. Due to a lack of self-healing function, mtDNA is usually degraded after being damaged [8, 23, 24], and new mtDNA is synthesized, in response to possible functional defects. For example, several mitochondrial alterations, which include changes in electron transport chain activity, increased oxidative stress and inadequate homeostasis responses, restore normal mitochondrial function and can trigger upregulation of the mtDNA replication machinery [5, 8, 25]. Notably, a dysfunction of the autophagy–lysosomal pathway in patients with CADASIL has been observed [26], which can inhibit degradation of mtDNA, leading to accumulation of damaged mtDNA in cells. Therefore, we speculate that an increase in mtDNAcn may be caused by the upregulation of mtDNA replication machinery or downregulation of mtDNA degradation [27].

In recent years, a leading hypothesis has been raised that epigenetic changes in mtDNA may be involved in the progression of neurodegenerative diseases [14]. In addition, in human specimens from patients with neurodegenerative disease or animal models of the disease, studies on methylation levels in mtDNA were almost always focused on the D-loop region [14]. As there is no intron in mtDNA, D-loop is the only noncoding mitochondrial region that regulates mtDNA replication and transcription, which makes D-loop a possible candidate for epigenetic modification [28]. In this study, we observed that there was a significant decrease in methylation levels of the mitochondrial D-loop region in peripheral blood of CADASIL patients, compared to controls. Importantly, a significant inverse association between D-loop methylation levels and mtDNAcn has been reported in different human samples, including peripheral blood [15, 16], placenta [29] and colorectal cancer [30, 31], suggesting that methylation levels of the D-loop regions may play a role in the regulation of mtDNA. Studies have attempted to determine whether hypomethylation of the D‑loop region is involved in regulation of the mtDNAcn [30, 31]. In colorectal cancer cell lines, artificially induced D-loop demethylation has led to increased mtDNAcn [30, 31], which consolidated the causal association between decreased D-loop methylation and increased mtDNAcn. Combined with previous studies [30, 31], our findings suggest that the increase in mtDNAcn in patients with CADASIL may be triggered by a decrease in D-loop methylation.

Finally, we attempted to elucidate the mechanism of mtDNAcn increase by mediation effect analysis and determined that decreased D-loop methylation partly mediated the association between increased mtDNAcn and occurrence of CADASIL. D-loop methylation likely regulates the transcription and translation of mtDNA, similar to what occurs in nuclear gene promoters [16]. Our current findings are similar to those of previous studies and provide epidemiological clues for the increase of mtDNA in patients with CADASIL. Due to limitations of medical records, some factors that may affect mitochondrial DNA methylation or mtDNAcn (e.g., body mass index, nutritional status and exercise data), were not adjusted as confounding factors in our study. Considering that the mtDNAcn is also controlled by many additional factors, including nuclear DNA and environmental exposures [32], whether there are other regulatory mechanisms for the increase of mtDNAcn in patients with CADASIL needs to be further explored. Further independent cohorts should be established or more methylation validation methods should be used in future studies.

## Conclusion

In conclusion, the present study identified a significant relationship between the increase of mtDNAcn and the occurrence of CADASIL, which was partially mediated by reduced D-loop methylation levels.

## Supplementary Information

**Additional file 1. Table S1.** The sequence of primer for mtDNAcn detection. **Table S2.** The associations between CADASIL and mtDNAcn in different adjusted models. **Table S3.** The associations between CADASIL and D-loop methylation levels in different adjusted models. **Table S4.** The associations between mtDNAcn and D-loop methylation levels in different adjusted models.

## Data Availability

All data generated or analyzed during this study are included in this published article and its supplementary information files.

## Abbreviations

CADASIL: Cerebral autosomal dominant arteriopathy with subcortical infarcts and leukoencephalopathy
mtDNAcn: Mitochondrial DNA copy number
VSMCs: Vascular smooth muscle cells
mtDNA: Mitochondrial DNA
ALS: Sporadic amyotrophic lateral sclerosis
AD: Alzheimer's disease
PD: Parkinson’s disease
PCR: Polymerase chain reaction
MTND1: Mitochondrial-encoded NADH dehydrogenase 1
MTND5: Mitochondrial-encoded NADH dehydrogenase 5
SAS: Statistical analysis system
