# Supplementary material for: Decreased mitochondrial D-loop region methylation mediates an increase in mitochondrial DNA copy number in CADASIL
Source: Clin Epigenetics. 2022 Jan 4;14:2. doi: 10.1186/s13148-021-01225-z (PMC8725280; doi:10.1186/s13148-021-01225-z)
Supplement: Supplementary file 1 — Additional file 1. Table S1. The sequence of primer for mtDNAcn detection. Table S2. The associations between CADASIL and mtDNAcn in different adjusted models. Table S3. The associations between CADASIL and D-loop methylation levels in different adjusted models. Table S4. The associations between mtDNAcn and D-loop methylation levels in different adjusted models. [file 13148_2021_1225_MOESM1_ESM.pdf]

Supplemental Material

Decreased mitochondrial D-loop region methylation mediates an increase in mitochondrial DNA copy number in CADASIL

Jiewen Zhang, Junkui Shang, Fengyu Wang, Xuejing Huo, Ruihua Sun, Zhixia Ren, Wan Wang, MiaoMiao Yang, Gai Li, Dandan Gao, Ruijie Liu, Pingping Bai, Shuyi Wang, Yanliang Wang, Xi Yan

Tables of Contents

Table S1. The sequence of primer for mtDNAcn detection

Table S2. The associations between CADASIL and mtDNAcn in different adjusted models

Table S3. The associations between CADASIL and D-loop methylation levels in different adjusted models

Table S4. The associations between mtDNAcn and D-loop methylation levels in different adjusted models

Table S1. The sequence of primer for mtDNAcn detection

**Name**	**Sequence (5'to3')**
SERPINA1 F	CAGTGAATAAATGAGGCGTACATCC
SERPINA1 R	GACTGTTTCTCATGCCTCTGGAAAG
SLCO2B1 F	CCTGATGCCTAGGTTTCTTTTCTTG
SLCO2B1 R	GGTCATCTGCCTACCCTAGAAC
MTND1 F	TACGGGCTACTACAACCCTTC
MTND1 R	ATGGTAGATGTGGCGGGTTT
MTND5 F	CATTACTAACAACATTTCCCCCGC
MTND5 R	GGCTGTGAGTTTTAGGTAGAGGG

Table S2. The associations between CADASIL and mtDNAcn in different adjusted models

	mtDNAcn	
	β(95%*CI*)	*P*
Total		
Model 1	-1.49(-2.08,-0.89)	<0.001
Model 2	-1.45(-2.04,-0.85	<0.001
Model 3	-1.45(-2.04,-0.85)	<0.001
Model 4	-1.48(-2.06,-0.90)	<0.001
Model 5	-1.48(-2.06,-0.90)	<0.001
Model 6	-1.46(-2.04,-0.87)	<0.001
Male		
Model 1	-0.90(-1.61,-0.18)	0.015
Model 2	-0.85(-1.57,-0.14)	0.019
Model 3	-0.88(-1.60,-0.16)	0.017
Female		
Model 1	-2.72(-3.70,-1.74)	<0.001
Model 2	-2.70(-3.68,-1.72)	<0.001
Model 3	-2.86(-3.88,-1.84)	<0.001

Model 1: adjust for age

Model 2: adjust for hypertension, diabetes mellitus, dyslipidemia

Model 3: adjust for age, hypertension, diabetes mellitus, dyslipidemia

Model 4: adjust for gender

Model 5: adjust for age, gender

Model 6: adjust for hypertension, diabetes mellitus, dyslipidemia

Table S3. The associations between CADASIL and D-loop methylation levels in different adjusted models

	D-loop methylation (%)	
	β(95%*CI*)	*P*
Total		
Model 1	38.44(31.48,45.39)	<0.001
Model 2	38.63(31.68,45.58)	<0.001
Model 3	38.61(31.66,45.57)	<0.001
Model 4	38.55(31.70,45.40)	<0.001
Model 5	38.57(31.73,45.41)	<0.001
Model 6	38.55(31.69,45.41)	<0.001
Male		
Model 1	33.92(25.20,42.63)	<0.001
Model 2	34.39(25.90,42.88)	<0.001
Model 3	34.41(25.80,43.02)	<0.001
Female		
Model 1	48.83(37.45,60.21)	<0.001
Model 2	46.44(35.09,57.78)	<0.001
Model 3	48.47(36.65,60.29)	<0.001

Model 1: adjust for age

Model 2: adjust for hypertension, diabetes mellitus, dyslipidemia

Model 3: adjust for age, hypertension, diabetes mellitus, dyslipidemia

Model 4: adjust for gender

Model 5: adjust for age, gender

Model 6: adjust for gender, hypertension, diabetes mellitus, dyslipidemia

Table S4. The associations between mtDNAcn and D-loop methylation levels in different adjusted models

	mtDNAcn	
	β(95%*CI*)	*P*
Total		
Model 1	-5.21(-7.30,-3.13)	<0.001
Model 2	-5.24(-7.31,-3.17)	<0.001
Model 3	-5.25(-7.32,-3.18)	<0.001
Model 4	-5.72(-7.79,-3.65)	<0.001
Model 5	-5.77(-7.84,-3.70)	<0.001
Model 6	-5.72(-7.78,-3.67)	<0.001
Male		
Model 1	-4.25(-6.99,-1.50)	0.002
Model 2	-4.31(-7.04,-1.58)	0.002
Model 3	-4.32(-7.04,-1.60)	0.002
Female		
Model 1	-7.64(-10.69,-4.58)	<0.001
Model 2	-7.74(-10.66,-4.83)	<0.001
Model 3	-7.65(-10.56,-4.74)	<0.001

Model 1: adjust for age;

Model 2: adjust for hypertension, diabetes mellitus, dyslipidemia;

Model 3: adjust for age, hypertension, diabetes mellitus, dyslipidemia;

Model 4: adjust for gender;

Model 5: adjust for age, gender;

Model 6: adjust for gender, hypertension, diabetes mellitus, dyslipidemia.
